# The additive value of complementing diagnostic idiopathic intracranial hypertension criteria by MRI – an external validation study

**DOI:** 10.1186/s10194-024-01781-8

**Published:** 2024-05-06

**Authors:** Stefan Macher, Wolfgang Marik, Nik Krajnc, Christoph Mitsch, Martin Michl, Nina Mueller, Klaus Novak, Sina Zaic, Christian Woeber, Berthold Pemp, Gabriel Bsteh

**Affiliations:** 1 Department of Neurology, Medical University of Vienna, Währinger Gürtel 18-20, Vienna, 1090 Austria; 2 Comprehensive Center for Clinical Neurosciences and Mental Health, Medical University of Vienna, Vienna, Austria; 3 Department of Neuroradiology, Medical University of Vienna, Vienna, Austria; 4 Department of Ophthalmology, Medical University of Vienna, Vienna, Austria; 5 Department of Neurosurgery, Medical University of Vienna, Vienna, Austria

**Keywords:** Idiopathic intracranial hypertension, Neuroimaging signs, Magnetic resonance imaging, Diagnosis, Criteria, Validation

## Abstract

**Background:**

Recently, diagnostic criteria including a standardized MRI criterion were presented to identify patients suffering from idiopathic intracranial hypertension (IIH) proposing that IIH might be defined by two out of three objective findings (papilledema, ≥ 25 cm cerebrospinal fluid opening pressure (CSF-OP) and ≥ 3/4 neuroimaging signs).

**Methods:**

To provide independent external validation, we retrospectively applied the proposed diagnostic criteria to our cohort of patients with clinical suspicion of IIH from the Vienna IIH database. Neuroimaging was reevaluated for IIH signs according to standardized definitions by a blinded expert neuroradiologist. We determined isolated diagnostic accuracy of the neuroimaging criterion (≥ 3/4 signs) as well as overall accuracy of the new proposed criteria.

**Results:**

We included patients with IIH (*n* = 102) and patients without IIH (no-IIH, *n* = 23). Baseline characteristics were balanced between IIH and no-IIH groups, but papilledema and CSF-OP were significantly higher in IIH. For the presence of ≥ 3/4 MRI signs, sensitivity was 39.2% and specificity was 91.3% with positive predictive value (PPV) of 95.2% and negative predictive value (NPV) 25.3%. Reclassifying our cohort according to the 2/3 IIH definition correctly identified 100% of patients without IIH, with definite IIH and suggested to have IIH without papilledema by Friedman criteria, respectively.

**Conclusion:**

The standardized neuroimaging criteria are easily applicable in clinical routine and provide moderate sensitivity and excellent specificity to identify patients with IIH. Defining IIH by 2/3 criteria significantly simplifies diagnosis without compromising accuracy.

**Supplementary Information:**

The online version contains supplementary material available at 10.1186/s10194-024-01781-8.

## Introduction

Idiopathic intracranial hypertension (IIH) is an increasingly prevalent syndrome of elevated intracranial pressure (ICP) of unclear etiology [1]. Due to a diverse clinical spectrum and the absence of a single pathognomonic feature, establishing diagnosis of IIH remains challenging [2]. Thus, diagnostic criteria have been revised several times with the modified Friedman criteria from 2013 currently broadly used [3]. Papilledema and elevated ICP remain unquestioned hallmarks of IIH along with normal cerebrospinal fluid (CSF) constitution and absence of structural lesions in neuroimaging [2–4].

However, the induction of a neuroimaging criterion has proven to create ambiguity in the field [3]. Revised Friedman criteria define presence of at least three out of four neuroimaging signs, i.e. (i) empty sella, (ii) flattening of the posterior aspect of the globe, (iii) distension of the perioptic subarachnoid space with or without a tortuous optic nerve and (iv) transverse sinus stenosis, as sufficient proof of elevated ICP to allow diagnosis of “suggested IIH without papilledema (IIH-WOP)” [3]. While this neuroimaging criterion seems to be reasonably specific for IIH, the relative rarity of IIH in relation to the high frequency of brain imaging performed in conditions mimicking IIH, e.g. primary headache disorders, combined with a high likelihood of accidental findings of these neuroimaging signs in a variety of neurological diseases has led to significant overdiagnosis of IIH [5–7]. This is aggravated by a lack of standardization and precise definitions underlying the neuroimaging criterion [8]. In an attempt to complement the revised Friedman criteria, Korsbaek et al. have provided standardized definitions of MRI signs of IIH and proposed that IIH diagnosis might be established if at least two out of three objective findings, i) papilledema, ii) ≥ 25 cm CSF opening pressure [OP] and iii) ≥ 3/4 MRI signs, i.e. moderate suprasellar herniation, distension of the perioptic nerve sheath, flattening of the globe and transverse sinus stenosis, are present [9].

The aim of this study was to apply these criteria retrospectively to a large cohort of patients with clinical suspicion of IIH to provide independent external validation.

## Methods

In this study we screened the Vienna-Idiopathic-Intracranial-Hypertension (VIIH) database, which includes both retrospectively and prospectively collected data, for patients referred to the Vienna specialized IIH outpatient clinic for clinical suspicion of IIH between 2014 and 2023 who had MRI at diagnosis available for reevaluation [10]. Exclusion criteria were pregnancy, breastfeeding, and previous IIH and/or secondary intracranial hypertension (IH).

The cohort was classified in patients receiving a diagnosis of IIH according to revised Friedman criteria (IIH-FC) and those not diagnosed with IIH (no-IIH-FC). IIH diagnosis was established by a standardized work-up as described in detail elsewhere [10].

The IIH-FC group was further divided into patients fulfilling revised Friedman criteria for definite IIH (def-IIH), probable IIH (prob-IIH, i.e. papilledema but CSF-OP < 25 cm H2O), IIH-WOP (no papilledema, CSF-OP ≥ 25 cm H2O, uni- or bilateral abducens nerve palsy) or suggested IIH-WOP (sug-IIH-WOP; no papilledema, no abducens nerve palsy, CSF-OP ≥ 25cmH2O, ≥ 3 IIH typical MRI signs) [3].

An experienced neuroradiologist (WM) blinded to diagnosis systematically reevaluated all neuroimaging scans applying the four MRI criteria as defined by Korsbaek et al. (Table 1): (i) suprasellar herniation graded from I-V with cut-off set at ≥ grade III, i.e. >1/3 of the sella height, in sagittal plane, (ii) uni- or bilateral distension of the perioptic nerve sheath > 2 mm in the coronal plane of T2 weighted images, measured 3 mm behind the globe, (iii) uni- or bilateral flattening of the globe by qualitative evaluation on axial T2 weighted images and (iv) bilateral transverse sinus stenosis by index of transverse sinus stenosis [ITSS] ≥ 4) [9, 11]. Minimal requirements for cerebral MRI sequences to evaluate pituitary and orbital morphology were set as defined by Korsbaek et al. (1.5T or 3T, at least T1 and T2w sequences in two different planes for excluding structural lesions, with or without thin-cut, fat-suppressed orbital sequences, venous non-contrast MR angiography or T1 post gadolinium sequence) [9].

**Table 1:** Koersbaek MRI criteria

MRI sign	Definition
Suprasellar herniation	I° no herniation II° mild herniation (< 1/3 of the sella height) III° moderate herniation (1/3–2/3 of the sella height) IV° severe herniation (> 2/3 of the sella height) V° enlarged sella turcica without observable pituitary parenchyma which was specified as < 1 mm of visible pituitary gland tissue
Distension of the perioptic subarachnoid space	was defined as > 2 mm distension of the perioptic subarachnoid space in the coronal plane of T2 weighted images
Flattening of the posterior aspect of the globe	was defined qualitatively uni- or bilateral in the axial plane (T2 weighted images)
Transverse venous sinus stenosis	was graded 1–4 on each side: I° ≤ 33% stenosis II° 33–66% stenosis III° ≥ 66% stenosis IV° hypoplasia or agenesia Hypoplasia was defined as the full-length diameter being < 1/3 of the superior sagittal sinus. The ITSS (index of transverse sinus stenosis) was calculated by multiplying the two grades and a value of ≥ 4 was considered patho- logical

### Statistics

Statistical analysis was performed using SPSS 26.0 (SPSS Inc, Chicago, IL, USA). Categorical variables were expressed in absolute frequencies and percentages, continuous parametric variables as mean and standard deviation (SD) continuous non-parametric variables as median with inter-quartile range (IQR) as appropriate depending on normal distribution assessed by Kolmogorov-Smirnov test.

Group comparisons were done by Fisher’s exact test, Chi-squared test, Mann-Whitney U test or Kruskal-Wallis-Test as appropriate. Significance level was set at *p* < 0.05.

Diagnostic accuracy was assessed by calculating sensitivity, specificity, positive and negative predictive value (PPV/NPV) for the presence of ≥ 3/4 neuroimaging signs in IIH-FC compared to no-IIH-FC by cross table analyses and area under the curve (AUC) by receiver operating characteristics (ROC) analyses. As an exploratory analyses, we also calculated diagnostic accuracy for discriminating subgroups of the IIH-FC group (def-IIH, prob-IIH, IIH-WOP, sug-IIH-WOP) from no-IIH-FC. Since diagnosis of sug-IIH-WOP is partly based on neuroimaging findings and including them could falsely inflate sensitivity, we conducted sensitivity analyses removing those patients from diagnostic accuracy calculations.

We further investigated if changing the suprasellar herniation cut-off from ≥ III° to ≥ V° or changing the transverse sinus stenosis criterion to any stenosis instead of ITSS ≥ 4 affects sensitivity and specificity of MRI criteria.

Lastly, we applied the proposed criteria as defined by Korsbaek et al. by reclassifying the whole cohort as IIH-KC if ≥ 2/3 criteria were fulfilled (papilledema, ≥ 25cmH2O CSF-OP and ≥ 3/4 MRI signs) or no-IIH-KC if ≤ 1/3 criteria were fulfilled [9]. To illustrate how the proposed criteria would affect IIH diagnosis, we also calculated the proportion of change of diagnosis in each subgroup (def-IIH, prob-IIH, IIH-WOP, sug-IIH-WOP, no-IIH) as defined by revised Friedman criteria.

### Ethics

The study was approved by the ethics committee of the Medical University Vienna (approval number: 2216/2020). As this is a retrospective analysis of data collected within clinical routine, the need for written informed consent from study participants was waived by the ethics committee. This study adheres to the reporting guidelines outlined within the ‘Strengthening the Reporting of Observational Studies in Epidemiology (STROBE) Statement.

## Results

**Fig. 1:**
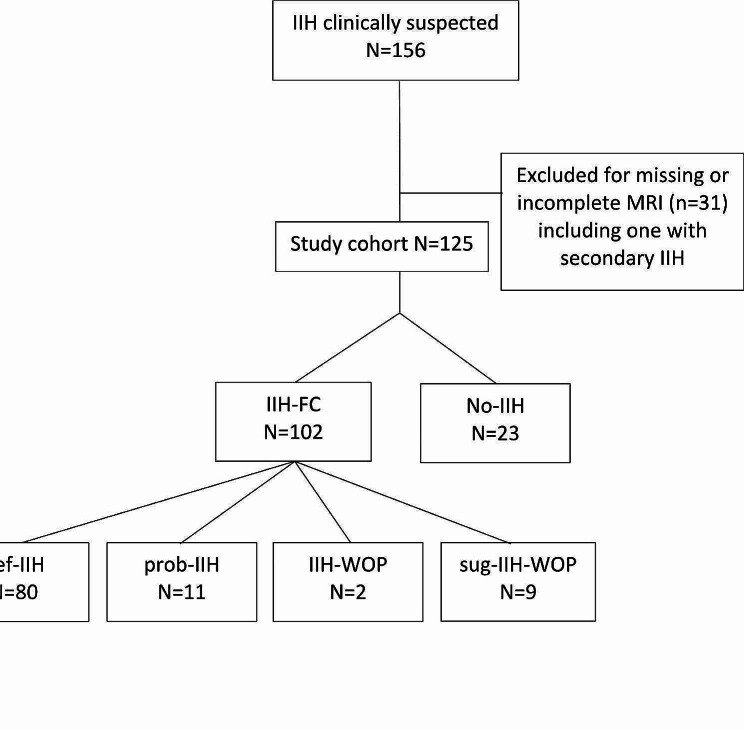
Inclusion protocol

From 156 patients screened from the VIIH database with suspicion of IIH, 31 had to be excluded due to unavailability of MRI at diagnosis with sufficient quality for reevaluation, of whom one patient was also excluded due to secondary IIH (see Fig. 1).

Of the 125 patients included, 102 (81.6%) fulfilled the revised Friedman criteria (IIH-FC), with 80 def-IIH, 11 prob-IIH, 2 IIH-WOP and 9 sug-IIH-WOP. Twenty-three patients received no diagnosis of IIH. Of those, 21 patients (91.3%) were diagnosed with primary headache disorder and two patients (8.7%) with a neuro-ophthalmological condition including pseudopapilledema and optic disc swelling.

Placeholder Fig. 1 Inclusion process.

Compared to the no-IIH-FC group, prevalence of papilledema (89.2% vs. 0%, *p* < 0.001) and elevated CSF-OP (30.5 cm H2O vs. 19 cm H2O, *p* < 0.001) was significantly higher in IIH-FC patients. Patients with prob-IIH had a significantly lower mean CSF-OP (22 cm H2O) than other IIH groups (*p* < 0.001). Per definition, all of the IIH-WOP patients and none of the sug-IIH-WOP patients had abducens nerve palsy, compared to 15.0% of def-IIH and none of prob-IIH patients. All non-IIH patients with swollen optic discs or abducens nerve palsy had CSF-OP < 25 cm H2O. A detailed description of group characteristics is given in Table 2.

**Table 2:** **Patient characteristics**

	IIH-FC^1^ (*n* = 102)	No-IIH (*n* = 23)	*p* value	def-IIH (*n* = 80)	prob-IIH (*n* = 11)	IIH-WOP (*n* = 2)	sug-IIH-WOP (*n* = 9)	*p* value
**Female (%)**	89.2%	87.0%	0.721^2^	92.5%	90.9%	50%	66.7%	0.069^4^
**Age (years, median, IQR)**	32.5 (29.2–36.7)	46.0 (20.5–52.5)	0.093^3^	30.0 (27.0-37.1)	28.0 (23.3–39.6)	34.1	33.0 (29.8–38.5)	0.384^5^
**BMI (median, IQR)**	32.7 (28.1–39.3)	27.9 (20.5–29.7)	0.269^3^	33.0 (30.7–38.4)	34.8 (27.0-39.3)	30.1	30.8 (27.9–38.3)	0.683^5^
**Papilledema (%)**	89.2	0%	< 0.001^2^	100%	100%	0%	0%	< 0.001^4^
**Headache (%)**	82.4%	91.3%	0.518^2^	81.8%	90.9%	100%	44.4%	0.053^4^
**Visual symptoms (%)**	80.4%	65.2%	0.120^2^	83.8%	72.7%	100%	66.7%	0.204^4^
**Tinnitus (%)**	37.1%	30.4%	0.594^2^	31.3%	72.7%	0%	66.7%	0.044^4^
**Abducens nerve palsy (%)**	13.7%	0%	0.121^2^	15.0%	0%	100%	0%	< 0.001^4^
**CSF-OP (cm H2O, median, IQR)**	30.5 (25.0–34.0)	19.0 (18.0-19.8)	< 0.001^3^	33.0 (26.0–34.0)	22.0 (19.0–23.0)	30.1	33.0 (30.8–34.5)	< 0.001^5^

^*1*^ *includes def-IIH, prob-IIH, IIH-WOP, sug-IIH-WOP. Def-IIH definite IIH; prob-IIH probable IIH; IIH-WOP IIH without papilledema; sug-IIH-WOP suggestive IIH without papilledema; BMI body mass index; CSF-OP cerebrospinal fluid opening pressure.* ^*2*^ *calculated by Fisher’s-Exact-Test.* ^*3*^ *calculated by Mann-Whitney-U-Test* ^*4*^ *calculated by Chi-Square Test.* ^*5*^ *calculated by Kruskal-Wallis-Test*

### Frequency and distribution of neuroimaging signs

Frequency of moderate suprasellar herniation (≥ grade III or more) was similar between the non-IIH and IIH-FC groups (47.8% vs. 48.9%, *p* = 0.929) as was suprasellar herniation grade V (17.4% vs. 18.7%; *p* = 0.886). Distension of the perioptic subarachnoid space (65.9% vs. 39.1%; *p* = 0.032) and any bilateral transverse sinus stenosis (71.4% versus 47.8%; *p* = 0.039) were significantly more frequent in the IIH-FC compared to the non-IIH cohort. Transverse sinus stenosis (ITSS ≥ 4) (40.7% vs. 26.1%, *p* = 0.619) and flattening of the posterior aspect of the globe (31.9% versus 13.0%; *p* = 0.116) were more common in the IIH-FC cohort, albeit not statistically significant.

The proportion of patients with suprasellar herniation ≥ III° was also similar between the subgroups (50.0% def-IIH, 55.6% sug-IIH-WOP and 45.5% prob-IIH; *p* = 0.712). Suprasellar herniation grade V appeared to be more frequent in patients with sug-IIH-WOP compared to def-IIH and prob-IIH (33.3% vs. 20.0% and 9.1%) without statistical significance. In the two patients with IIH-WOP there was no evidence of empty sella grade III or higher. Distension of the perioptic nerve sheath, posterior globe flattening and transverse sinus stenosis (ITSS ≥ 4 or any) were more frequent in the sug-IIH-WOP cohort (100%, 77.8%, 77.8% and 100%) compared to def-IIH (71.3%, 35.0%, 36.3% and 76.3%) or prob-IIH (27.3%, 9.1%, 18.2% and 36.3%, see Table 3).

**Table 3:** Distribution of neuroimaging signs among patients with FC-IIH, no-IIH and subgroups

	IIH-FC with papilledema^1^ (*n* = 91)	No IIH (*n* = 23)	*p* value*	def-IIH (*n* = 80)	prob-IIH (*n* = 11)	IIH-WOP (*n* = 2)	sug-IIH-WOP (*n* = 9)
Suprasellar herniation ≥ III°	48.9%	47.8%	0.929	50.0%	45.5%	0%	55.6%
Suprasellar herniation V°	18.7%	17.4%	0.886	20.0%	9.1%	0%	33.3%
ONSD	65.9%	39.1%	0.032	71.3%	27.3%	50%	100%
PGF	31.9%	13.0%	0.116	35.0%	9.1%	0%	77.8%
TSS (ITSS ≥ 4)	40.7%	26.1%	0.619	36.3%	18.2%	0%	77.8%
Any TSS	71.4%	47.8%	0.039	76.3%	36.3%	0%	100%

ONSD optic nerve sheath distension, PGF posterior globe flattening, TSS transverse sinus stenosis. *2calculated by Fisher’s-Exact-Test.

### Diagnostic accuracy of ≥ 3/4 neuroimaging signs in identifying IIH

In the overall cohort, the ≥ 3/4 neuroimaging sign criterion distinguished IIH-FC and no-IIH with 39.2% sensitivity, and 91.3% specificity resulting in a diagnostic accuracy of 65.2% (Table 3).

Looking at discriminative ability in the subgroups, overall diagnostic accuracy as well as sensitivity/specificity remained similar when leaving out patients with sug-IIH-WOP from the IIH cohort and when comparing only patients with papilledema (def-IIH and prob-IIH) with no-IIH (Table 3). However, separating prob-IIH from the no-IIH group by ≥ 3/4 neuroimaging signs, sensitivity (18.2%) and consequently accuracy (54.7%) dropped significantly.

Presence of ≥ 3/4 neuroimaging signs predicted the correct diagnosis in 95.2% of IIH-FC and 94.1% of def-IIH, as well as in 94.4% of IIH-FC when removing patients with sug-IIH-WOP and in 94.4% of IIH-FC when removing patients without papilledema (Table 4). The negative predictive value of the neuroimaging criterion was low overall, but 91.3% in distinguishing IIH-WOP from no-IIH patients.

**Table 4:** Diagnostic accuracy of ≥ 3/4 neuroimaging signs in identifying IIH.

	Sensitivity	Specificity	PPV	NPV	AUC
IIH-FC vs. no-IIH	39.2%	91.3%	95.2%	25.3%	0.652
IIH-FC without sug-IIH-WOP vs. no-IIH	36.6%	91.3%	94.4%	26.3%	0.638
IIH-FC with papilledema (def + prob) vs. no-IIH	38.2%	91.3%	94.4%	26.9%	0.642
def-IIH vs. no-IIH	39.5%	91.3%	94.1%	30.4%	0.655
prob-IIH vs. no-IIH	18.2%	91.3%	50.0%	70.0%	0.547
IIH-WOP vs. no-IIH	0%	91.3%	0%	91.3%	0.457

Looking into the impact of changing the suprasellar herniation cut-off from ≥ III° to ≥ V°, we found that sensitivity was reduced from 39.2 to 24.5%, while specificity remained at 91.3% and PPV slightly decreased to 92.6% (vs. 95.2%) (see Supplemental Table 1).

When changing the transverse sinus stenosis criterion to any stenosis instead of ITSS ≥ 4, sensitivity increased from 39.2 to 48.0% but specificity dropped to 78.3% (vs. 91.3%) and PPV to 90.7% (vs. 95.1%) (see Supplemental Table 2).

Using a suprasellar herniation cut-off ≥ V° combined with any transverse sinus, sensitivity was lowered to 36.3% with specificity at 82.6% (see Supplemental Table 2).

### Impact of diagnosing IIH based on 2/3 diagnostic criteria

**Fig. 2:**
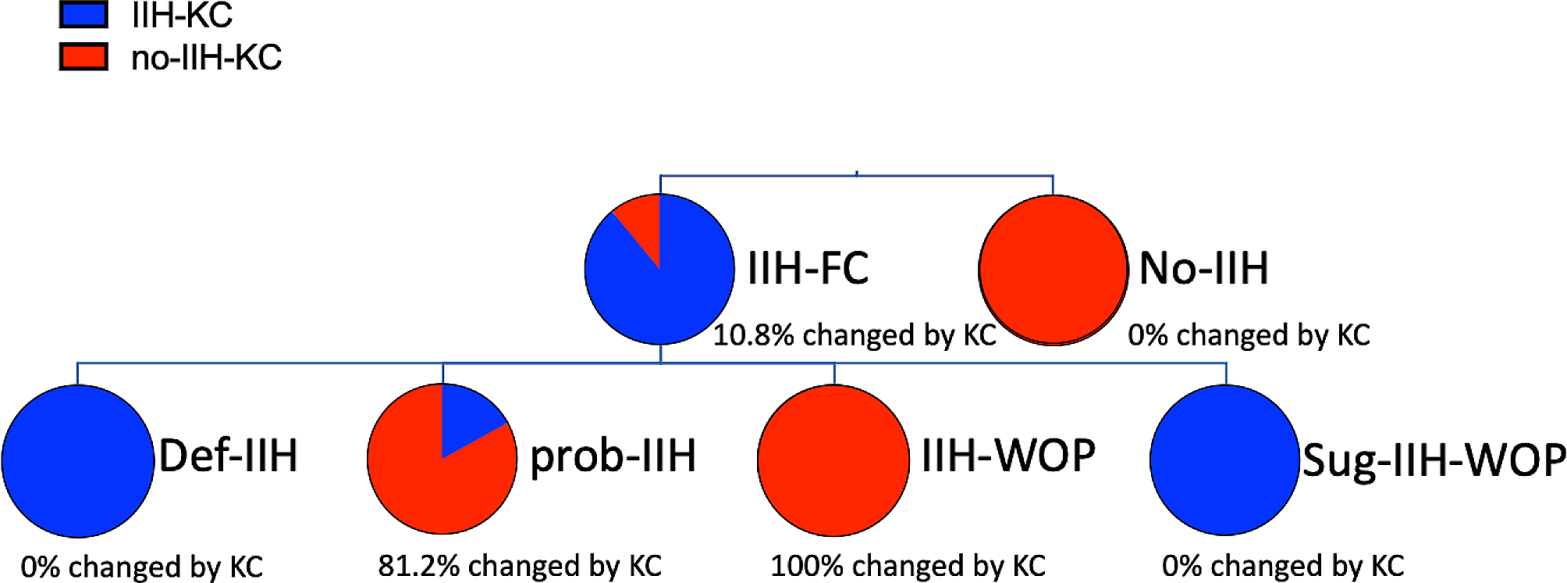
Impact of proposed new IIH criteria on diagnosis of IIH.

Reclassifying the whole cohort by applying the proposed criteria by Korsbaek (KC), we found that all patients without IIH according to revised Friedman criteria (FC) remained diagnosed no-IIH (100%) and all patients with def-IIH according to FC also received a diagnosis of IIH with KC (100%). Overall, 89.2% received a diagnosis of IIH with both FC and KC (see Fig. 2). The main change in classification occurred in patients receiving a diagnosis of probable IIH and IIH-WOP according to FC, of whom only 18.8% and 0%, respectively, were diagnosed as IIH when applying KC (Fig. 2).

## Discussion

Currently, diagnosis of IIH is widely established by applying the revised Friedman criteria 2013 [3]. While those have contributed a great deal in terms of providing a common platform in clinical routine and science, some issues remain unresolved. Foremost, the positive usage of MRI for identifying intracranial hypertension beyond just ruling out secondary causes lacked standardized definitions and was limited to patients without papilledema. Recently, Korsbaek et al. provided standardized definitions of MRI signs and suggested an updated version of IIH diagnostic criteria, which use the neuroimaging criterion (≥ 3 neuroimaging signs) on equal terms as papilledema and CSF-OP ≥ 25 cm H2O requiring two of three to be present for diagnosis of IIH [9].

Here, we applied the Korsbaek criteria on a large and well-characterized independent cohort of patients with and without IIH as external validation. Encouragingly, we found high specificity (91.3%) and PPV (95.2%) of ≥ 3/4 neuroimaging signs for identifying IIH, providing confirmative evidence for the applicability and value of these standardized diagnostic criteria in clinical practice without increasing risk of misdiagnosis. Applying the suggested updated version of IIH diagnostic criteria, 100% of patients without IIH as well as 100% of patients with definitive IIH were correctly identified.

The high specificity and PPV of the neuroimaging criterion reported in the original study was confirmed in our cohort. With respect to 100% accuracy of the updated criteria in patients without IIH, this is particularly encouraging as the rate of IIH misdiagnosis is high and incidental findings of neuroimaging signs are reported in up to 49% of patients undergoing brain MRI without clinical suspicion of IIH [5, 7]. On the other hand, MRI signs of IIH are often underestimated and partly overcalled by less experienced neuroradiologists in clinical routine, which is likely due to insufficient training but also imperfect definitions of MRI signs [12]. Hence, a high level of precision is required, which precludes the use of isolated elevated OP or one to two neuroimaging signs as diagnostic for IIH. In that regard, we also looked into the impact of changing the definitions for suprasellar herniation from partial (≥ III°) to complete (≥ V), whereby specificity was not increased but sensitivity was significantly lowered. Of note, we observed a rather high proportion of patients with suprasellar herniation, distension of the perioptic subarachnoid space and transverse venous sinus stenosis in our No-IIH group. However, the rates are comparable to the only other study applying the same standardized MRI criteria to their non-IIH cohort as well as within the range of the reported prevalence of incidental MRI findings in patients without papilledema [5, 9]. Although empty sella/suprasellar herniation is the most well-known imaging feature of IIH, it does not reliably distinguish between IIH and non-IIH in isolation [9].

When considering any signs of transverse sinus stenosis instead of requiring ITSS ≥ 4, sensitivity was only slightly increased at the cost of a considerable drop-off in specificity. Thus, the neuroimaging criterion as defined in the updated criteria seems to provide an optimal balance between specificity and sensitivity.

Since decades, the handling of patients with papilledema but CSF-OP < 25 cm H2O (probable IIH as per revised Friedmann criteria 2013) and those without papilledema but CSF-OP ≥ 25 cm H2O (IIH-WOP and suggested IIH-WOP) continues to create controversy in the field regarding their actual meaning and pathological value [13]. IIH-WOP is probably rare with a prevalence of < 6% among patients with IIH, and even lower in unselected patients with chronic headache [10, 14, 15]. Incorrect assessment of papilledema and low disease awareness may lead to underestimation of IIH, but potentially also to overdiagnosis of IIH-WOP [5, 7]. With Korsbaek criteria, patients with “suggested IIH-WOP” according to revised Friedman criteria would all receive a diagnosis of IIH, driven by the neuroimaging criterion substituting for the absence of papilledema. In return, the majority of patients with “probable IIH” and “IIH-WOP” according to revised Friedman criteria would not be diagnosed with IIH by Korsbaek criteria as they do not fulfill the neuroimaging criterion and, thus, only one of three criteria. While Korsbaek criteria obviously do not clarify diagnosis in all cases, the incorporation of standardized MRI signs provides means to simplify diagnosis of IIH, which will increase usability for clinicians and may reduce misdiagnosis. Still, there remains an area of uncertainty, a “grey area” of patients with 1–2 MRI signs of IIH and/or CSF-OP between 20 and 24 cm H2O. Further studies as well as novel diagnostic biomarkers are therefore urgently needed.

Importantly, it needs to be emphasized that the positive use of MRI as an indicator of elevated intracranial pressure does not by any means replace the need for lumbar puncture in diagnostic work-up of IIH. The Friedman criteria as well as the Koersbak criteria include a normal CSF composition as mandatory for establishing IIH diagnosis in order to rule out differential diagnoses. Hence, every patient suspected to have IIH is required to undergo LP and every institution diagnosing IIH needs to be able to perform ICP measurement.

Strengths and Limitations.

The strengths of this study is the in terms of IIH large number of patients with comprehensive characterization and work-up [10]. However, the number of patients without IIH as well as the number of patients with IIH-WOP and suggested IIH-WOP was still too small to enable valid subgroup analyses and, thus, the distribution of MRI signs of IIH may not be representative of other cohorts. Of note, the VIIH cohort includes a lower proportion of secondary IIH than usual, which is due to the reference conditions for assessment within our special outpatient clinic requiring exclusion of obvious causes of secondary IIH (primarily sinus venous thrombosis) in advance. Thus, our results may not be completely generalizable to cohorts with higher proportions of secondary IIH.

MRI imaging in our cohort was performed during diagnostic work-up; however, it should be critically noted that there was no strictly standardized interval. Also, MRI scans were performed on different scanners with varying image acquisition protocols. Specifically, optic nerve sheath distension and posterior globe flattening were evaluated on T2-weighted images since thin cut fat-suppressed orbital sequences, which allow more robust detection and may therefore increase sensitivity, were not available for a sufficient number of patients to include them in the analyses. Finally, all MRI assessments were performed by a senior neuroradiologist with extensive experience in IIH imaging (WM). Thus, diagnostic accuracy may be lower in a real-world setting with less experienced raters and a less detailed work-up.

## Conclusion

In conclusion, we can confirm that the standardized neuroimaging signs reported by Korsbaek et al. are applicable in clinical routine and provide moderate sensitivity and excellent specificity to identify patients with IIH.

The suggested update of the Friedman criteria incorporating those standardized neuroimaging signs combined with papilledema and CSF-OP ≥ 25 cm H2O by using a two out of three requirement simplifies diagnosis without compromising accuracy.

### Electronic supplementary material

Below is the link to the electronic supplementary material.

Supplementary Material 1

Supplementary Material 2

## Data Availability

No datasets were generated or analysed during the current study.
