# Supplementary material for: The additive value of complementing diagnostic idiopathic intracranial hypertension criteria by MRI – an external validation study
Source: J Headache Pain. 2024 May 6;25(1):70. doi: 10.1186/s10194-024-01781-8 (PMC11071194; doi:10.1186/s10194-024-01781-8)
Supplement: Supplementary file 1 — Supplementary Material 1 [file 10194_2024_1781_MOESM1_ESM.docx]

|  | Sensitivity | Specificity | PPV | NPV | AUC |
| --- | --- | --- | --- | --- | --- |
| IIH-FC vs.  no-IIH | 24.5% | 91.3% | 92.6% | 21.4% | 0.577 |
| IIH-FC without sug-IIH-WOP vs.  no-IIH | 21.5% | 91.3% | 90.9% | 22.3% | 0.561 |
| IIH-FC with papilledema (def+prob) vs.  no-IIH | 22.0% | 91.3% | 90.9% | 22.8% | 0.563 |
| def-IIH vs.  no-IIH | 25.0% | 91.3% | 90.9% | 25.9% | 0.578 |
| prob-IIH vs.  no-IIH | 100% | 91.3% | 84.6% | 100% | 0.457 |
| IIH-WOP vs.  no-IIH | 0% | 91.3% | 0% | 91.3% | 0.457 |

Supplemental table 1 Diagnostic Accuracy of ≥3/4 neuroimaging signs with suprasellar herniation ≥V° instead of ≥III° in identifying IIH (ITTS≥4)
