# Supplementary material for: The additive value of complementing diagnostic idiopathic intracranial hypertension criteria by MRI – an external validation study
Source: J Headache Pain. 2024 May 6;25(1):70. doi: 10.1186/s10194-024-01781-8 (PMC11071194; doi:10.1186/s10194-024-01781-8)
Supplement: Supplementary file 2 — Supplementary Material 2 [file 10194_2024_1781_MOESM2_ESM.docx]

|  | Sensitivity | Specificity | PPV | NPV | AUC |
| --- | --- | --- | --- | --- | --- |
| ES III° | | | | | |
| IIH-FC vs.  no-IIH | 48.0% | 78.3% | 90.7% | 25.4% | 0.631 |
| IIH-FC without sug-IIH-WOP vs.  no-IIH | 45.2% | 78.3% | 89.4% | 26.1% | 0.617 |
| IIH-FC with papilledema (def+prob) vs.  no-IIH | 46.2% | 78.3% | 89.4% | 26.9% | 0.622 |
| def-IIH vs.  no-IIH | 50.0% | 78.3% | 88.9% | 31.0% | 0.641 |
| prob-IIH vs.  no-IIH | 18.2% | 78.3% | 28.6% | 33.3% | 0.482 |
| IIH-WOP vs.  no-IIH | 0% | 78.3% | 0% | 10.0% | 0.391 |
| ES V° | | | | | |
| IIH-FC vs.  no-IIH | 36.3% | 82.6% | 90.2% | 22.6% | 0.593 |
| IIH-FC without sug-IIH-WOP vs.  no-IIH | 32.3% | 82.6% | 89.4% | 23.2% | 0.572 |
| IIH-FC with papilledema (def+prob) vs.  no-IIH | 33.0% | 82.6% | 88.2% | 23.8% | 0.576 |
| def-IIH vs.  no-IIH | 36.3% | 82.6% | 87.9% | 27.1% | 0.593 |
| prob-IIH vs.  no-IIH | 9.0% | 82.6% | 20.0% | 65.5% | 0.458 |
| IIH-WOP vs.  no-IIH | 0% | 82.6% | 0% | 90.5% | 0.413 |

Supplemental table 2 Diagnostic Accuracy of ≥3/4 neuroimaging signs with suprasellar herniation ≥III° and ≥V° in identifying IIH (using any sinus stenosis instead of ITTS≥4)
